# Homeward

**DOI:** 10.1089/pmr.2021.0032

**Published:** 2022-04-04

**Authors:** Julia K. Nguyen

**Affiliations:** Kaiser Permanente, Panorama City Medical Center, Home Infusion Pharmacy, Panorama City, California, USA.

I've heard that it's the young deaths that kill you.

JC was 17 years old, a native son of California. At 5 foot 9 inches and 210 lbs, he was a growing oak, with leaves green and vibrant from every angle, with the promise of mightiness. But in the towering shadows of outspread branches, a voracious beetle (chondrogenic osteosarcoma) was boring through the bark and into the concentric rings of the wood underneath. After undergoing a hemipelvectomy and assault by chemotherapy and then radiation, JC learned that the silent invader had proliferated and advanced into his femoral, sacral, and pelvic bones.

We had taken care of him for two months. This included providing parenteral nutrition, and the usual care that would be expected to stay the effects of the well…you know…the thing that should not be named. He was treated until he was admitted for bacterial and fungal sepsis at a local Children's Hospital in June. When he returned to us in February, he was on hospice care for intractable pain. I knew it was the beginning of the end.

His initial intravenous morphine 10 mg/h patient-controlled analgesia had escalated from 40 mg/hr to 80 mg/hr to 100 mg/hr to 300 mg/hr in just 10 days. He was completely bedridden and also on a dartboard of medications.^†^ Although JC received temporary relief from multiple boluses and could find sleep in fleeting moments, the effects did not last for more than two to three hours at a time. He would wake up screaming, shaking, and jerking in excruciatingly sharp shooting pain emanating from his hip, pelvis, and back. His pain was aggravated by movement and quantified using the Visual Analog Scale as being 7/10 to 10/10 most of the time. The disease was silent no more.

His mother sobbed on the phone, “No mother should have to outlive her child and see so much pain.” It was so hard on her, a single mom trying to make ends meet. She was so exhausted down to her roots. JC was angry at the world and depressed in the moment. The puppy he had received as a birthday gift to welcome him home had just died of “kennel cough.” She needed JC's grandmother to help with the caregiving. I remember her choked-up voice before she hung up, “Please help me, please help us.”

That was it, a simple plea for a not-so-simple situation.

I called the hospice physician who was relieved to hear from me. She had JC's mother's permission to control his pain at any cost. Could we buy time with a trial of decreasing the morphine dosing, hoping for a possible opioid-induced hyperalgesia during this tense environment? She hesitated. “It's going to be a hard sell; mom is expecting more, not less. And we will be in the same situation, or worse if it does not work as planned.”

“How about opioid rotation to intravenous hydromorphone?” I asked. “No go…he had hallucinations prior to discharge home,” she responded. I groaned inwardly knowing there was no easy solution. In our experience with end-stage illness and extreme dosing, the line between disease progression and opioid tolerance was often blurred. I proceeded down my mental checklist. He was on adjuvants, check. But even with antimetics he was having nausea that limited his ability to regularly ingest medications.

My shoulder muscles tightened.

“How much time do you think he has left?” I tentatively asked. “He's young, with good cardiac function, but bowel movements have decreased to every five days. His will to live is fading fast.” Add ketamine, I thought. Y-site not tested; and the admixture was intravenous (IV) compatible. He had a double lumen peripherally inserted central catheter (PICC), and his other line was occupied by parenteral nutrition. What concentration would I compound to make it work? Did I need another peripheral or subcutaneous line? Would that be enough to work quickly? My mind raced to keep up with my pounding heart. I took a deep breath to calm my heartbeat and reminded myself to focus.

I offered up a ketamine–fentanyl–midazolam (KFM) cocktail letting her know that our local team had no previous actual experience with this off-label approach. I felt like Hermione Granger conjuring from the Book of Potions. Any purported outcomes were based on medical literature in hospitalized older adults of variable dosing ranges and testimonials from other acute medical oncology centers.^1^ The consensus was that managing bone cancer pain in the home was a beast that enraged unique inflammatory and worsening neurological changes. We understood this battle. It was a fight not to save JC from dying young but a fight to honor his dying well.

JC's physician, nurse, and I held a teleconference to plan the initiation of the cocktail, scheduled for the following morning. Overnight, after-hours staff on call had increased his morphine to 500 mg/h. At 10:00 in the morning, the nurse started ketamine 2 mg/mL–fentanyl 5 mcg/mL–midazolam 0.1 mg/mL (KFM) combination through a CADD^®^ Prizm pump. The starting dose was 10 mL/h with 5 mL every 15 minutes as needed for breakthrough pain. We decreased morphine by 100 mg/h concurrently and nursing assessed him every hour with orders to call the physician to titrate to comfort.

The effects were immediate. By the next day at 2:00 pm, morphine was discontinued. JC was comfortable and responding well to boluses. The involuntary jerking had stopped and he only had some pain when turning. Methadone, lorazepam, and haloperidol were held. For the next nine days, we titrated the KFM. I took all calls related to JC's care and raced to keep abreast of providing the short-term stability medication. I wanted to stay ahead of our pharmacy's supply given the current background of national supply chain-related shortages.

Multiple one-liter bags were delivered three times a week. I was concerned about the volume of fluid being provided and recommended separating the triple mix for improved stability and ease, titrating the ketamine separately. She agreed. We would use the Y-site for the ketamine and infuse the fentanyl–midazolam with another pump. We had limited stability data for concentrating the cocktail so parenteral nutrition volume was decreased.

At first, JC was able to sleep more peacefully for only a few hours. Gradually the duration of his sleep increased until he was able to sleep for the whole night within five days of starting KFM. JC no longer screamed. He no longer moaned. There was no more grimacing, no more restlessness, and no more agitation. His grandmother reported to me, “He has nothing to complain about!” JC was on ketamine 120 mg/h/fentanyl 300 mcg/h/midazolam 6 mg/h when I received the last phone call. It had been 16 days after initiation, for a total of 26 days under our care. His nurse reported, as a small measure of success, that he was finally able to enjoy a popsicle before going to sleep.

When he passed away, his face was peaceful. His grandmother and mother were grateful, with tears streaming down their faces, and thankful for the nurse at the home and thankful for all that had been done for JC. I exhaled deeply.

As I drove home in a black sea of blood red taillights, the glimmering night stars were obscured by billowy clouds. Cool moist air blew hair against my face. I felt the sensation, but I was numb. What was I feeling, sorrow or joy? By naming the fear, would it loosen its grip on me? What had transpired? Experience was a brutal task master, tasting salty and bittersweet at the same time. The slight humming buzz in my head grew louder like a street market of voices jostling to be heard before erupting. JC had given me knowledge as a gift in exchange of his life. We were bound inextricably through the unveiling of this mysterious cosmic eternal truth.

We all are comprised of physical matter and to exist without hope is a bleak prospect. How do we answer the call to matter to others during our time? Although JC's story occurred over 15 years ago, it was a transformative experience that nearly crushed me as a new practitioner. Triumph of hope can be a blinding impetus for therapeutic interventions. And so at what point might a clinician decide that the alleviating powers of medications have reached their limit?

Our medical center has since developed evidence-based guidelines to navigate those necessary conversations for home palliative management of intractable agitation and suffering and malignant bowel obstruction. Moreover, our team has elected to voluntarily participate in medical aid in dying as legalized in California. Although the optimism connected to new therapies is sometime unsustained, clearly we can explore therapies previously abandoned as too aggressive or invasive as they may provide succor for death and dying in the home. JC's legacy is a reminder of the value of death and for me, continues to fuel the beacon for performance improvement in end-of-life care.^2^

## Disclaimer

All views expressed by the author are solely her opinion and do not reflect the opinions of SCPMG or Kaiser Permanente. SCPMG has not reviewed this submission and makes no representation regarding its accuracy or completeness.

## Abbreviation Used

KFM: ketamine–fentanyl–midazolam

## Notes

^†^
 Fentanyl, oral hydromorphone, methadone, oxycodone, oxymorphone, haloperidol, pregabalin, nortriptyline, dexamethasone, lorazepam, and quetiapine.
